# Comparison of two puncture methods in the implantation of totally implantable venous access ports: a retrospective study

**DOI:** 10.3389/fsurg.2025.1696009

**Published:** 2025-12-09

**Authors:** Kun Fang, Chang-Jia Li, Jia-jun Zhang, Yu-Kun Liu

**Affiliations:** 1 Breast Surgery Department, Breast Disease Center, Qingdao Central Hospital, University of Health and Rehabilitation Sciences, Qingdao, China; 2 Department of Colorectal and Anal Surgery, Qingdao Central Hospital, University of Health and Rehabilitation Sciences, Qingdao, China; 3 Outpatient Department, Qingdao Central Hospital, University of Health and Rehabilitation Sciences, Qingdao, China

**Keywords:** totally implantable venous access ports, puncture methods, internal jugular vein, bleeding volume, appearance of the incision

## Abstract

**Background:**

Totally implantable venous access ports (TIVAPs) are widely used for cancer patients requiring long-term chemotherapy. The classical procedure of vein puncture in the implantation of TIVAPs is to puncture by a needle and then make a skin incision to insert a guidewire and a dilator. The purpose of this study is to compare the effects of making a skin incision before vs. after TIVAP operation on the occurrence of complications.

**Methods:**

Patients with breast cancer were from the Affiliated Qingdao Central Hospital of Qingdao University. The participants were randomly assigned in to two groups with different puncture methods in the implantation of TIVAPs. The difference between two puncture methods were assessed by length of incision, procedure time, bleeding volume, extent of pain and appearance satisfaction after procedure. Multivariable linear regression was used to investigate the difference of complications between groups.

**Results:**

A total of 300 patients with breast cancer were included in our study. The mean age of participants was 53.47 years. No significant differences were found between groups in length of incision, procedure time, and extent of pain. However, participants who received the incision before internal jugular vein puncture had less bleeding volume (*p* = 0.04) and were more satisfied with the appearance of their incision (*p* = 0.03).

**Conclusion:**

We present a method that makes the incision before vein puncture for guidewire and dilator insertion in TIVAD placement. This method may result in less bleeding and improved appearance for patients undergoing chemotherapy for cancer.

## Introduction

Long-term venous access devices are important for administration of repeated intravenous antineoplastic therapy (1). Due to the safety and reliability, totally implantable venous access ports (TIVAPs) which allow accessing a large vein by a port placed into a subcutaneous pocket have been widely used for patients requiring long-term chemotherapy after breast cancer surgery (2–4). TIVAPs can be implanted via several veins including the subclavian vein and external or internal jugular vein (5, 6). Though TIVAP was considered a feasible and safe way for long-term chemotherapy (7), some possible complications may occur related to the implantation technique and maintenance of TIVAP (8, 9). Pneumothorax and hematoma may occur rarely during reservoir implantation due to injury to adjacent structures (10, 11). However, pain and bleeding were inevitable during port implantation.

In the classical procedures of port implantation, a skin incision was performed after the vein was punctured under ultrasound guidance to insert a guidewire and a dilator with a peel-away sheath (12). But the punctured needle may hinder the cutting of the skin. In this study, we tried to make the incision before the vein was punctured for guidewire and dilator insertion to reduce the complications and aimed to compare the two different puncture methods used in the implantation of totally implantable venous access ports in a sample of 300 patients with breast cancer who received TIVAPs implantation.

## Materials and methods

### Study participants

A total of 300 patients with breast cancer who received TIVAPs implantation were included from the Affiliated Qingdao Central Hospital of Qingdao University from June 2018 to June 2021. All the TIVAPs were implanted through the internal jugular vein. The exclusion criteria include the following: (1) coagulation abnormalities; (2) the TIVAPs were implanted through the veins except internal jugular vein; (3) the diameter of internal jugular vein at the punctured side is less than 8 mm; (4) other contraindications of TIVAP implantation. Demographic information of included patients was collected by an electronic medical record system. The participants were classified into two groups according to the different puncture methods used in the puncture of the internal jugular vein.

The study was conducted in accordance with the Helsinki declaration, and the protocol for this study was approved by the Institutional Ethics Committee of the Affiliated Qingdao Central Hospital of Qingdao University. Written informed consent was obtained from all participants.

### Implantation procedure

Surgery was performed under local anesthesia. Patients lay supine with their heads turned to the other side of the puncture point (13). A puncture point of internal jugular vein was marked firstly with the high-frequency ultrasound probe (14). In group A, the internal jugular vein was punctured by a puncture needle and then an incision was cut to insert a guidewire and a dilator with a peel-away sheath. In group B, the incision was cut before puncture to insert a guidewire and a dilator. The port and the catheter were introduced after the successful puncture in both groups. A skin incision was cut for the port on the anterior of the thorax and a pouch was created by blunt dissection. The port was fixed at the pectoral fascia. And the catheter was inserted through a subcutaneous channel into the dilator. The TIVAP was standardized tested after wound closure. A schematic figure to clearly demonstrate the procedural differences in Supplementary Figure S1.

### Assessment of complications

The complications were assessed by the length of incision, procedure time, bleeding volume, extent of pain and appearance satisfaction after procedure. The length of incision was measured at a week after the procedure. Bleeding volume was quantified by weighing gauze pieces. The extent of pain was assessed by the numerical rating scale (range from 0 to 10, 1 = no pain and 10 = maximum imaginable pain). Appearance satisfaction was assessed by patients themselves at six weeks after the procedure. The patients rate their appearance satisfaction of the incision by a number ranging from 0 to 10. No serious adverse reactions were observed during the follow-up, and all patients completed the study.

### Statistical analysis

The differences of demographics and clinical characteristics between groups were analyzed with Wilcoxon test or chi square test. The differences of length of incision, procedure time, bleeding volume, extent of pain and appearance satisfaction after procedure were analyzed using multivariable linear regression adjusted for age. statistical analysis was carried out using R version 3.5.3.

## Results

### Demographic and clinical characteristics of included participants

A total of 300 participants with breast cancer were enrolled in this study. The participants were randomly assigned to group A and group B with 150 participants in each group. The demographic and clinical characteristics of the participants were summarized in Table 1. In brief, the mean age of the participants in group A was 53.87 ± 9.70 years. The mean age of the participants in group B was 53.07 ± 10.41 years old. No significant difference was found in the age between two groups (*p* = 0.49). A hundred and nine participants were punctured at left internal jugular vein. A hundred and ninety-one participants were punctured at the right internal jugular vein. No differences were found in the puncture sides between two groups as well.

**Table 1:** Characteristics of subjects in this study.

Characteristic	All participants	Group A	Group B	*p* value
No.	300	150	150	-
Age (years)	53.47 ± 10.05	53.87 ± 9.70	53.07 ± 10.41	0.49
Puncture side (*n*, %)				0.15
Left	109 (36.3%)	48 (32.0%)	61 (40.7%)	0.119
Right	191 (63.7%)	102 (68.0%)	89 (59.3%)	
Complications				
Length of incision (mm)	9.14 ± 2.23	9.25 ± 1.93	9.03 ± 2.50	0.39
Procedure time (min)	27.35 ± 4.51	27.20 ± 4.49	26.80 ± 3.80	0.10
Bleeding volume (mL)	16.89 ± 10.99	18.20 ± 11.43	15.58 ± 10.40	0.04
Extent of pain	1.90 ± 0.99	1.84 ± 0.99	1.96 ± 0.98	0.27
Appearance satisfaction	8.18 ± 0.90	8.07 ± 0.87	8.29 ± 0.92	0.03

**Figure 1:**
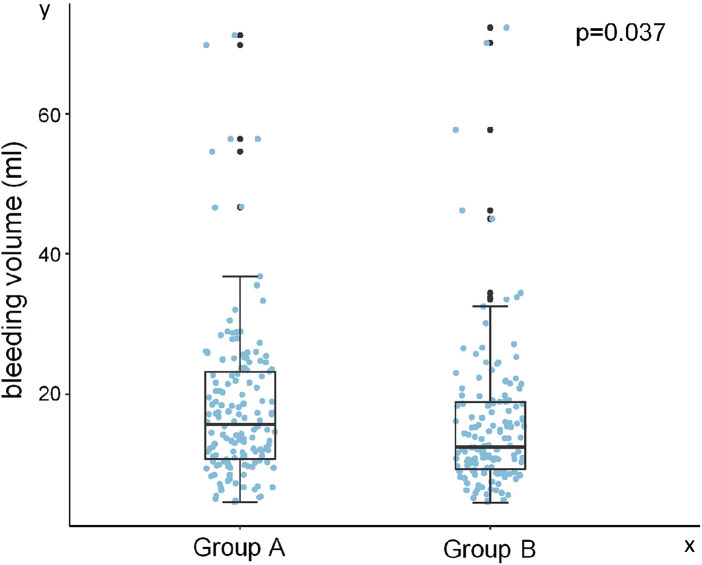
Bleeding volume comparison between Group A and Group B. Group A: the internal jugular vein was punctured by a puncture needle and then an incision was cut to insert a guidewire and a dilator with a peel-away sheath; Group B: the incision was cut before puncture to insert a guidewire and a dilator.

**Figure 2:**
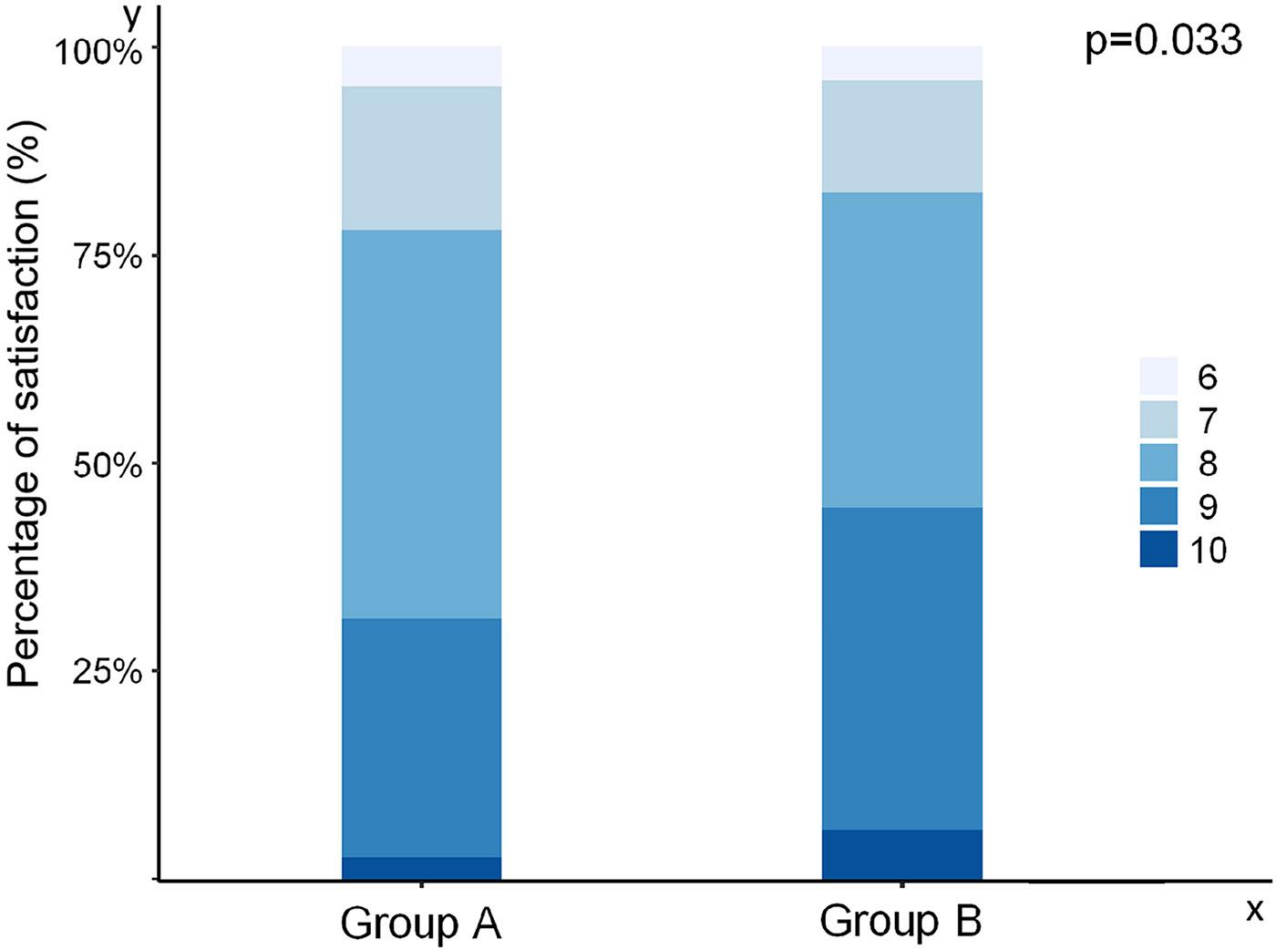
Satisfactory about the appearance of incision compared Group A with Group B. Group A: the internal jugular vein was punctured by a puncture needle and then an incision was cut to insert a guidewire and a dilator with a peel-away sheath; Group B: the incision was cut before puncture to insert a guidewire and a dilator.

All participants underwent the procedures successfully without any major complications, though minor issues like arterial puncture, multiple punctures, and hematoma occurred. In the comparation of length of incision, procedure time, bleeding volume, extent of pain and appearance satisfaction after procedure between two groups. We did not find any differences in length of incision, procedure time and extent of pain between two groups (Table 1). However, participants who received internal jugular vein puncture before the incision was cut had less bleeding volume (*p* = 0.04, Figure 1) and more satisfactory about the appearance of their incision (*p* = 0.03, Figure 2).

## Discussion

In this study, we included 300 patients aimed to investigate the differences of two methods used in the puncture of internal jugular vein in the implantation of TIVAPs with respect to patient pain perception, length of incision, procedure time, bleeding volume and appearance satisfaction after procedures. We have found that patients whose incision was cut before vein puncture had less bleeding volume and were more satisfactory about their appearance of the incision.

TIVAP implantation is a commonly performed procedures for patients required long-term chemotherapy (15, 16). Previous studies have been performed to identify a favorable implantation method. Subclavian vein and internal jugular vein were both considered safe for catheter insertion (17–19). The internal jugular vein puncture might be better regarding pain perception, radiation dose, postinterventional catheter tip position and port function (17, 20). In addition, the safety can be increased under fluoroscopy or ultrasound guided especially when the internal jugular vein is used (21). Subclavian vein puncture is performed more often when ports are typically implanted below the clavicle. Thus, ultrasound-guided puncture via the internal jugular vein was used widely for catheter placement (22, 23). In our study, the TIVAPs of all patients were successfully implanted using the internal jugular vein approach with no severe complications. Pain and bleeding were inevitable during the puncture of internal jugular vein though no severe complications were occurred. In our study, the bleeding volume was increased when the internal jugular vein was punctured before the incision cutting. This might because puncture the internal jugular vein first means bleeding time is relatively longer. The mean procedure time was short in the group of patients whose incision was cut before vein puncture, though no significant difference was found in the procedure time between two groups. Besides, the punctured needle may hinder the cutting if the needle was punctured into the vein first. Therefore, the incision might not be neat. This may be the reason why patients in group B were more satisfactory about the appearance of the incision.

In clinical practice, intravenous ports are preferably placed on the right side due to the shorter and less convoluted route of the right-side vessels (24). The insertion length is also shorter compared to the left side, resulting in a lower incidence of complications. Therefore, if possible, the right side is chosen. Since the breasts are bilaterally symmetric organs, if a mastectomy for breast cancer has been performed on the right side, an intravenopus port is generally not placed on the same side of the chest wall, necessitating the choice of the contralateral side. Thus, the decision between the left and right sides is not random. This study excluded this influencing factor, further validating the reliability and robustness of the results. More randomized controlled trials are needed in the future to further validate the results of this study.

A strength of this study is the high number of patients in the sample and all procedures. Nonetheless, the research has its limitations. As an observational study, it faces issues like retrospective data collection, insufficient documentation, inherent biases, and restricted control over confounding factors. Also, the fact that the study was done at just one institution might limit the extent to which the results can be generalized to other patient groups and practice scenarios (25).

In conclusion, we present a method that make the incision before vein puncture for guidewire and dilator insertion in TIVADs placement. This method might be better with less bleeding volume and better appearance for patients treated with chemotherapy for cancer.

## Data Availability

The raw data supporting the conclusions of this article will be made available by the authors, without undue reservation.
