# Supplementary material for: Comparison of two puncture methods in the implantation of totally implantable venous access ports: a retrospective study
Source: Front Surg. 2025 Dec 9;12:1696009. doi: 10.3389/fsurg.2025.1696009 (PMC12722802; doi:10.3389/fsurg.2025.1696009)
Supplement: Supplementary file 1 [file Supplementaryfile1.docx]

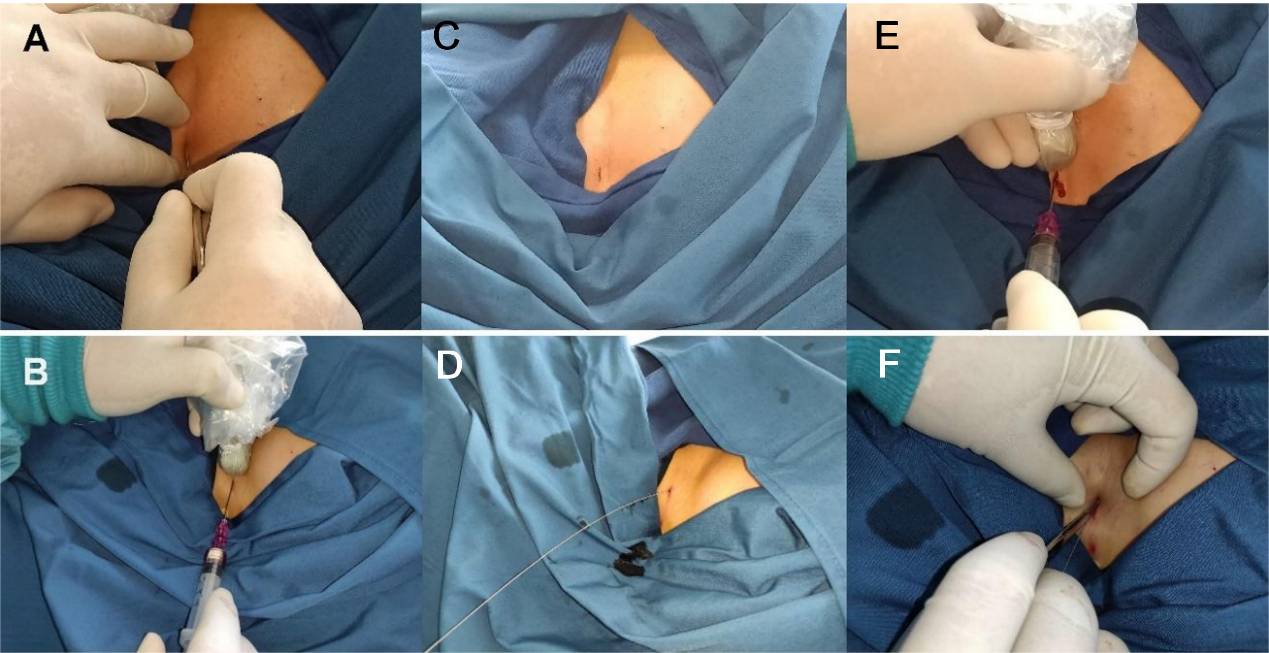


Figure S1. A schematic figure to clearly demonstrate the procedural differences. (A, C, E) The incision was cut before puncture to insert a guidewire and a dilator. (B, D, F) The internal jugular vein was punctured by a puncture needle and then an incision was cut to insert a guidewire and a dilator with a peel-away sheath.
